# Dysphagia: A Case Report of an Atypical Presentation of Statin-Induced Necrotizing Myositis

**DOI:** 10.7759/cureus.43587

**Published:** 2023-08-16

**Authors:** Michaela B Polmann, Richard I Suarez, Ali Saad, Kebir H Bedran

**Affiliations:** 1 Herbert Wertheim College of Medicine, Florida International University, Miami, USA; 2 Pathology, University of Miami Miller School of Medicine, Miami, USA; 3 Hospital Medicine, Baptist Health South Florida, Miami, USA

**Keywords:** statin use, proximal weakness, elevated creatine phosphokinase (cpk), statin-induced necrotizing autoimmune myositis, rare cause of dysphagia

## Abstract

Statin medications act by inhibiting the enzyme hydroxy-3-methylglutaryl coenzyme A (HMG-CoA) reductase (HMGCR), thus decreasing hepatic cholesterol synthesis. They are considered the mainstay treatment of hypercholesterolemia due to their tremendous efficacy and mortality benefit. Although generally well tolerated, statins may adversely affect skeletal muscle resulting in side effects ranging from mild myalgia to life-threatening necrotizing myositis. Statin-induced necrotizing autoimmune myositis is a rare yet devastating adverse effect that may occur shortly after initiation of therapy or after several years of use. Unfortunately, medication discontinuation has shown no impact on prevention or alleviation of symptoms. Though there is currently no definitive guidance for the treatment of this condition, corticosteroids are generally considered to be first line, via high-dose oral prednisone or intravenous methylprednisolone. In this case report, we discuss the case of a 72-year-old male with an unusual presentation of statin-induced necrotizing autoimmune myositis: dysphagia, weakness, and weight loss. His diagnosis was confirmed by muscle biopsy indicating necrotizing myositis and his serum was found to be strongly positive for anti-HMG-CoA reductase antibodies. This patient had a very brief history of statin use, but his primary care provider discontinued the medication a couple of months prior to symptom onset due to elevated liver function tests. He was treated with aggressive intravenous fluid hydration and intravenous corticosteroids during an extended inpatient hospital stay. He was discharged to a rehabilitation facility. This report demonstrates the importance of creating a wide differential for patients who present with fatigue, generalized weakness, and dysphagia. It is essential to always consider statin-induced necrotizing myositis if a patient has a history of statin use, even if the statin has been discontinued. Necrotizing myositis demands timely diagnosis and management to improve mortality.

## Introduction

Statin medications act by inhibiting the enzyme hydroxy-3-methylglutaryl coenzyme A (HMG-CoA) reductase (HMGCR), thus decreasing hepatic cholesterol synthesis. They are the mainstay of treatment of hypercholesterolemia due to tremendous efficacy and mortality benefit. However, their use is met with challenges due to potential side effects including myalgia, myopathy, rhabdomyolysis, necrotizing myositis, liver disease, liver failure, and peripheral neuropathy [1]. Myopathy is the most common cause of statin discontinuation [2]. Necrotizing autoimmune myositis is a rare complication that may occur due to the development of immunoglobulin G (IgG) autoantibodies against HMGCR [1,3]. This autoimmune phenomenon, unlike other immune-mediated drug toxicities, typically does not improve upon medication discontinuation and requires extensive immunosuppressive therapy [4].

The identification of necrotizing myositis secondary to statin usage demands timely diagnosis and management to improve mortality. In the literature, case reports and other research articles have described the typical presentation of necrotizing myositis secondary to statin use as severe muscle weakness and/or myalgia in multiple muscle groups that persist despite statin discontinuation [3,5,6]. However, it is important to be aware of atypical presentations of this severe adverse event. In this case report, we discuss an atypical presentation of statin-induced necrotizing autoimmune myositis: a patient with chief complaints of dysphagia, weakness, and weight loss.

## Case presentation

A 72-year-old Brazilian-American man with a past medical history of type II diabetes mellitus, hyperlipidemia, and hypertension presented to the emergency department with a four-to-six-week history of dysphagia to solids, resulting in approximately 20 pounds of weight loss. He also had progressively worsening proximal muscle weakness and fatigue. He denied muscle pain, fever, symptoms of upper respiratory tract infection, chest pain, shortness of breath, diarrhea, constipation, and emesis. He also denied alcohol, tobacco, and illicit drug use. He was employed in the construction industry and was able to perform physical labor and activities of daily living prior to the onset of these symptoms. The patient had previously taken atorvastatin (10mg then 20mg) for approximately 8-10 months, but it was discontinued by his primary care provider two months prior to presentation to the hospital due to elevated liver enzymes without symptoms of myositis. He was also previously taking metformin, glipizide, lisinopril, and hydrochlorothiazide for his chronic conditions, however, with his recent weight loss he no longer required these medications. He was not on any medications at the time of admission.

The physical exam was notable for proximal muscle weakness worse in the lower extremities, but no focal neurologic deficits. The patient appeared cachectic and dehydrated. There was no tenderness to muscle palpation and no skin rash. Vital signs upon admission were stable and unremarkable. Table 1 summarizes the laboratory studies performed, including liver function tests, autoimmune studies, and inflammatory markers. The results were significant for elevated alanine transaminase (ALT) (534 U/L), aspartate aminotransferase (AST) (415 U/L), creatine kinase (CK) (10,406 U/L), aldolase (107.5 U/L), and positive anti-HMG-CoA reductase antibodies. All other results were unremarkable.

**Table 1:** Patient laboratory values Significant values are bolded. ALT: alanine transaminase, AST: aspartate transaminase, ALP: alkaline phosphatase, CK: creatine kinase, ESR: erythrocyte sedimentation rate, CRP: C-reactive protein, ANA: anti-nuclear antibody, ASMA: anti-smooth muscle antibody, WNL: within normal limits

Laboratory Test	Result
ALT	534 U/L (elevated)
AST	415 U/L (elevated)
ALP	WNL
Bilirubin	WNL
Hepatitis Panel (A, B, C)	Negative
Alpha 1 Antitrypsin	WNL
Ceruloplasmin	WNL
CK	10,406 U/L (elevated)
Aldolase	107.5 U/L (elevated)
ESR	WNL
CRP	WNL
ANA	Negative
ASMA	Negative
Anti-Mitochondrial Antibody	Negative
Anti-Smith Antibody	Negative
Anti-Jo-1 Antibody	Negative
Anti-SNRP Antibodies	Negative
Anti-Gliadin IgG and IgA	Negative
Tissue Transglutaminase IgG and IgA	Negative
IgA Endomysial Antibodies	Negative
Anti-HMG-CoA Reductase Antibodies	159 (strongly positive)

Chest radiography, non-contrast computerized tomography of the chest, and non-contrast computerized tomography of the abdomen/pelvis were unremarkable. A complete abdominal ultrasound showed normal liver parenchyma and no evidence of cholelithiasis. Liver Doppler ultrasound showed portal and hepatic veins patent with appropriate directional flow. Endoscopic biopsies showed mild active chronic *Helicobacter pylori* gastritis and duodenal mucosa with mild acute and chronic inflammation. A barium swallow study showed moderate oropharyngeal dysphagia with a prolonged oral stage. On one occasion, there was penetration of thin liquid to the cords with no attempt to clear the residue and suspected aspiration. Multiple swallows were noted during the study, however, performance gradually declined with subsequent trials, and it was suspected that fatigue impacted swallow performance.

**Figure 1:**
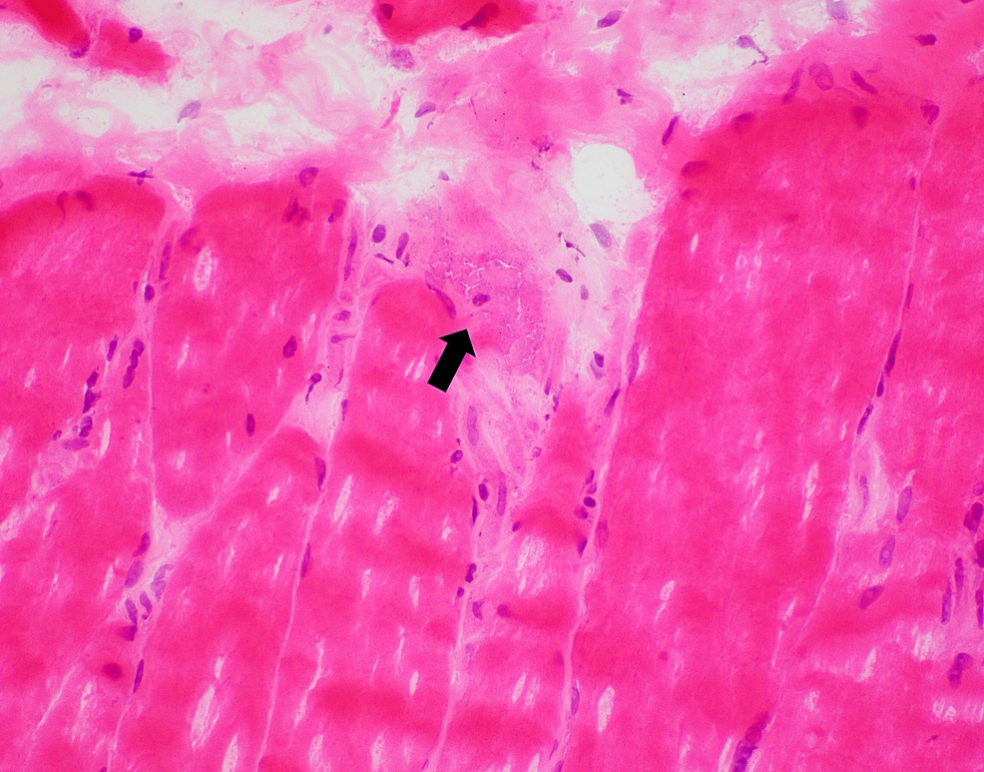
Muscle biopsy with necrotic muscle fiber

**Figure 2:**
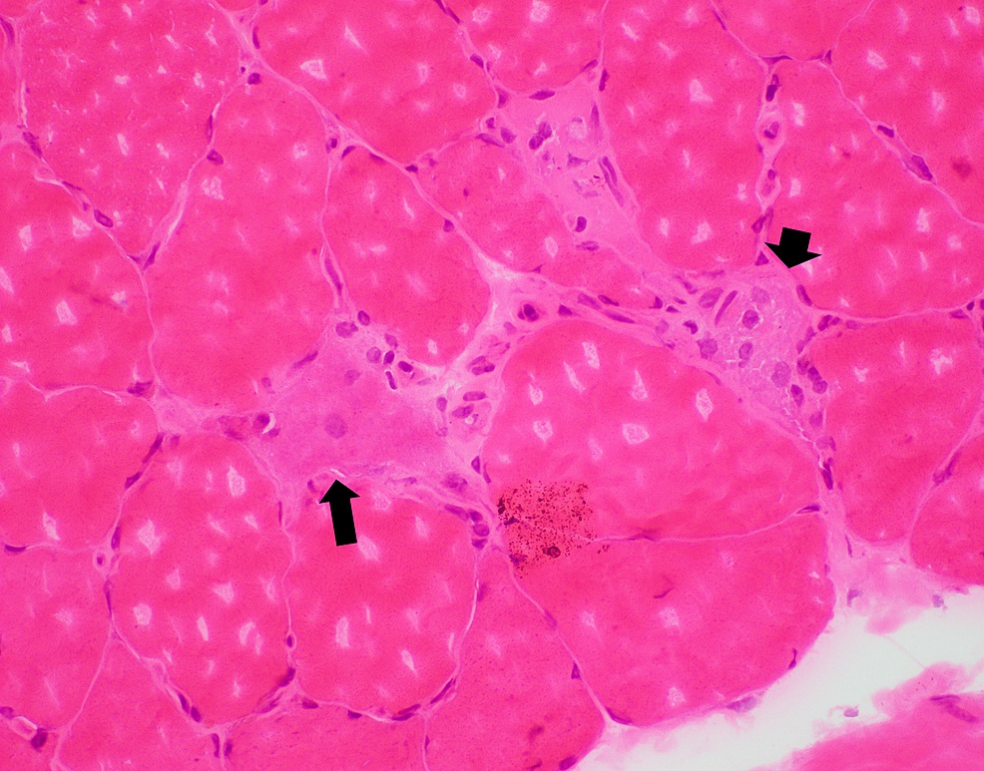
Muscle biopsy with degenerating muscle fiber and myophagocytosis The long arrow indicates degenerating/regenerating muscle fiber. The short arrow indicates myophagocytosis.

Histologic examination of a muscle biopsy from the right thigh shows chronic inflammatory infiltrate present around individual fibers. The inflammatory cells consisted exclusively of T-lymphocytes and macrophages as demonstrated by immunostains with CD3 and CD68, respectively. Numerous necrotic fibers are present (Figure 1). Myophagocytosis is readily identified, and scattered degenerating/regenerating fibers are noted (Figure 2). No vasculitis or granulomas identified.

Upon discovery of significantly elevated CK and aldolase levels, the patient began receiving intravenous fluid hydration and intravenous methylprednisolone. He reported gradual improvement in generalized weakness and fatigue. Liver enzymes improved to an ALT of 349 U/L and AST of 194 U/L. Twenty-three days after the initial CK measurement, the CK had dropped to 2,345 U/L. Considering the patient’s presentation, history, and diagnostic results, including muscle biopsy and positive anti-HMG-CoA reductase antibodies, he was diagnosed with necrotizing myositis secondary to statin usage. The patient was discharged to an acute rehabilitation facility on oral prednisone with a follow-up appointment with rheumatology to further titrate immunosuppressive therapy. With rehabilitation and corticosteroids, the patient showed significant improvements in strength and overall functional status six months after discharge from the hospital. He reported subjective improvement in dysphagia back to his baseline prior to this diagnosis.

## Discussion

When we examine generalized weakness as a presentation, many diagnoses appear on the differential, but this case can serve as a reminder about the importance of considering uncommon pathologies. Patients with myopathies, regardless of cause, classically present with signs of weakness, which is often chronic. There are various reasons patients develop myopathies, including by immune-mediated mechanisms. This group of myopathies is characterized by autoimmune disorders that result in skeletal muscle inflammation and myofiber necrosis with minimal inflammatory infiltrates on biopsy [5,4]. These immune-mediated necrotizing myopathies (IMNM) tend to have elevated CK - a marker of muscle destruction - a lack of extra-muscular involvement and often more severe disease [5,4]. IMNM are difficult to distinguish clinically but are sub-classified into three main categories as it relates to their antibodies: anti-signal recognition (SRP), anti-3-hydroxy-3-methylglutaryl-CoA reductase (HMGCR), and antibody-negative [3]. Patients diagnosed with IMNM as a whole are more often women, however, those with positive HMGCR antibodies are more often men and more often carry the DRB1:11 alleles [7]. Understanding a patient’s history, alongside physical exams and other diagnostic evaluations remains key to being able to diagnose these more uncommon causes of usual symptoms.

Statin-induced necrotizing autoimmune myositis is a rare yet devastating adverse effect of statin use estimated to occur in every two or three out of 100,000 patients treated with statins [8]. The most common clinical presentation is bilateral proximal muscle weakness [9]. The average age of presentation is approximately 65 years old [9]. It may occur shortly after initiation of therapy or after several years of use. Interestingly, our patient did not develop symptoms during statin use, but rather two months after he discontinued the medication due to elevated liver enzymes, which further supports the notion that discontinuation does not prevent nor improve symptoms. Cases with a delayed presentation have been reported, but most patients present with symptoms while taking a statin [10,11]. The reasoning for delayed presentation is unclear, but may perhaps be due to genetic susceptibility. Furthermore, our patient only took a statin for approximately three months, compared to an average of approximately 40 months of statin use in most patients who develop statin-induced necrotizing autoimmune myositis [9].

Typical laboratory presentation includes markedly elevated CK levels, with nearly 90% of cases having levels over 2000 IU/L [8]. Individuals with symptoms of weakness and prior statin use who have positive anti-HMC-CoA reductase autoantibodies are strongly suspected of having the condition. There have been few reports of dysphagia as the presenting symptom for this condition, although dysphagia is commonly seen in patients with idiopathic inflammatory myopathies including dermatomyositis, inclusion body myositis, and polymyositis [12]. In these cases, dysphagia is caused by the inflammatory involvement of muscles involved in swallowing. This is likely a similar etiology to the dysphagia in the patient presented in this case. Dysphagia increases the risk of aspiration pneumonia and is associated with increased mortality [12]. This case report demonstrates the importance of investigating statin-induced necrotizing autoimmune myositis as a cause for dysphagia in patients with current or prior statin use and elevated creatine kinase.

There is currently no definitive guidance for treatment of this condition. However, corticosteroids are generally considered to be first line, via high-dose oral prednisone or intravenous methylprednisolone. Additional treatments include intravenous immunoglobulin (IVIG) or other immunosuppressive agents such as azathioprine and methotrexate [3]. Early treatment may improve prognosis and therefore prompt diagnosis is essential. Response to therapy varies greatly between patients, with a small subset of patients improving without treatment, but the majority requiring immunosuppressive therapy. In rare and aggressive cases, patients may not respond to a single immunosuppressive medication and may require treatment with rituximab or plasmapheresis. In some cases, muscle strength may not be regained [13,9]. Some patients will not fully respond to medical treatment and the condition can prove fatal due to respiratory failure secondary to diaphragmatic weakness [14]. However, most patients do experience full symptom resolution after treatment [9].

## Conclusions

Fatigue and weight loss are common reasons for patient presentation to either the outpatient clinic or the emergency department, especially when associated with other, more alarming symptoms like chest pain, nausea, weakness, persistent diarrhea, and dysphagia. Statin-induced necrotizing autoimmune myositis is a rare adverse effect of statin that classically presents with proximal muscle weakness and significantly elevated CK. Improvement is typically not seen with statin discontinuation and therefore immunosuppressive therapy is often warranted. Dysphagia is an uncommon presentation of this condition but can increase the risk of morbidity and mortality by predisposing the patient to aspiration pneumonia. A wide differential diagnosis and prompt evaluation for necrotizing autoimmune myositis is prudent if a patient presents with dysphagia and a history of statin use.
